# Effects of Frozen Storage Time, Thawing Treatments, and Their Interaction on the Rheological Properties of Non-Fermented Wheat Dough

**DOI:** 10.3390/foods12234369

**Published:** 2023-12-04

**Authors:** Jingjie Yang, Yingquan Zhang, Jikai Jiang, Bo Zhang, Ming Li, Boli Guo

**Affiliations:** 1 Institute of Food Science and Technology, Chinese Academy of Agriculture Sciences/Comprehensive Utilization Laboratory of Cereal and Oil Processing, Ministry of Agriculture and Rural Affairs of the People Republic of China, Beijing 100193, China; janeyang95@126.com (J.Y.); zhangyingquan@caas.cn (Y.Z.); 15736938676@163.com (J.J.); zjzb1978@126.com (B.Z.); mingli@caas.cn (M.L.); 2 Western Agriculture Research Center, Chinese Academy of Agricultural Sciences, Changji 831100, China

**Keywords:** frozen non-fermented dough, thawing treatment, frozen storage, dough rheological properties

## Abstract

In this study, the effects of frozen storage time, thawing treatments, and their interaction on the rheological properties of non-fermented dough were evaluated. Texture profile analysis (TPA), rheological measurements, including strain/frequency sweep, and creep-recovery measurement were applied to the dough. Compared with unfrozen fresh dough, the frozen storage time (S) and thawing treatment (T) influenced almost all indicators significantly, and their mutual effects (S × T) mainly affected the hardness and springiness. Frozen time was the main factor resulting in the destruction of non-fermented dough during the thawing treatments. Moreover, refrigerator thawing (4 °C) produced a dough with minimal changes in the rheological properties, regardless of the frozen storage time. Meanwhile, microwave thawing resulted in lower G′ and lower zero shear viscosity (*η*_0_) values, as well as higher maximum creep compliance (*J_max_*) and hardness values. Moreover, the difference between the three thawing treatments was exacerbated after 30 days of frozen storage. SEM images also showed that long-term frozen storage combined with microwave thawing seriously destroyed the rheological properties, structural stability, and inner microstructure of the dough.

## 1. Introduction

Recently, the demand for frozen non-fermented dough products such as noodles, steamed bread, and dumplings in the Chinese market has increased because of its economic superiority [1]. However, the quality of frozen dough products is often inferior compared with fresh ones because freezing and frozen treatments can destroy the dough’s structure [2]. Lots of work has been conducted to investigate the relationship between the quality degradation of frozen dough and freezing parameters such as freezing temperature [3,4,5], frozen storage duration [6,7,8], and freeze−thaw cycles [9,10]. Furthermore, some experts have explored methods to promote the quality of frozen dough by using various improvers [11,12].

Additionally, some researchers have stated that the influence of thawing treatments on the quality of frozen dough should also be taken into consideration as a key parameter as the thawing process is an essential procedure for some frozen dough before cooking [13]. The dough’s textural properties and cooking performance might be influenced by the thawing conditions. For example, Du et al. [14] found that fermented sweet dough thawed at 35 °C with an RH of 85% had the overall best quality. In addition, the microwave-thawed dough tended to have a harder texture, and refrigerator-thawing produced dough with lower resistance against mixing [13]. In our previous study, we proved that thawing treatments had a notable impact on the structural properties of frozen dough. Refrigerator (4 °C) thawing produced a dough with a softer texture, whereas microwave thawing mostly worsened the dough’s quality. However, almost no studies have focused on the rheological properties of long-term frozen storage dough treated with different thawing treatments. Whether the negative impact of different thawing treatments on the textural properties of dough remain constant after long-term frozen storage is still unclear.

Rheological properties are other key parameters used to evaluate the quality of dough, which is also widely used to predict the quality of final products and to adjust processing conditions in the production of frozen dough products [15]. Rheological properties can be characterized by the results of dynamic strain sweeps, frequency sweeps, temperature sweeps, and creep-recovery tests [16,17,18,19,20,21]. However, to our knowledge, no much literature has evaluated the interaction effects between frozen storage and thawing treatments on the viscoelastic properties of non-fermented dough.

Hence, this study aims to evaluate the effects of frozen storage time, different thawing treatments, and their interaction on the rheological properties and textural properties of non-fermented dough. For this purpose, TPA analysis and rheological measurements, including strain sweep and frequency sweep, and creep-recovery testing were applied using different instrumental analyses. Meanwhile, the dough’s internal structure was observed by scanning electron microscopy (SEM) directly. The current study will provide new insight into the textural properties and rheological properties of the dough treated with frozen storage and thawing. It also provides theoretical support for reducing the freezing-induced dough deterioration from the perspective of optimizing the frozen-thawing technology.

## 2. Materials and Methods

### 2.1. Wheat Flour

The flour material was produced from the winter wheat variety Xiaoyan 22. The milling process was performed based on AACCI Method 26-21.02. The basic information about the flour is provided in the Table 1.

### 2.2. Dough Preparation

#### 2.2.1. Dough Mixing and Freezing

To prepare the dough, 300 g of raw material was placed into the farinograph. The mixing process was completed until the dough reached a consistency of 500 Brabender Units (BU). Subsequently, the dough was cut into 60 g portions and sealed in bags. The packaged dough was then moved into an ultra-low temperature refrigerator for freezing. The freezing temperature was set as −30 °C, and the freezing process continued until the core temperature of the dough decreased to −18 °C. Afterward, the frozen dough was stored at −18 °C for 1 day and 30 days, respectively. The fresh dough that reached to 500 BU were regarded as the control samples (CK).

#### 2.2.2. Thawing Treatments

Frozen dough was thawed under various thawing conditions, and the thawing process continued until the center temperature of the dough increased to 4 °C. Three thawing treatments were applied: RT-thawing in a refrigerator at 4 °C for 6 h, CT-thawing in an environmental chamber at 25 °C and RH 85% for 1 h, and MT-thawing in a microwave oven at 1000 W for 25 s [22].

### 2.3. Measurement of Dough’s Rheological Properties

#### 2.3.1. Dynamic Strain Sweep

The linear viscoelasticity region (*LVR*) of the dough was measured by dynamic strain sweep. Based on previous methods [23], each dough sample (2 g) was placed between two plates 25 mm in diameter, which were loaded onto the rheometer, and the excess dough was scraped off. The measurement involved a 10 min relaxation period followed by dynamic strain sweep testing at 25 °C. The testing conducted with strain ranged from 0.01% to 100% and had an angular frequency of 10 s^−1^.

#### 2.3.2. Dynamic Frequency Sweep

The frequency sweep tests were conducted as described by Yang et al. [7] to obtain *G*′ and *G*″ as a function of the frequency. Testing was conducted from an angular frequency of 0.1 to 100 s^−1^ at a strain of 0.1%. To obtain the values of *z*′ and *K*′, the frequency sweep data were fitted to the power law model as follows:

$$G\prime \;=K\prime {\left(\omega \right)}^{z\prime }$$

where ω is the angular frequency.

#### 2.3.3. Creep-Recovery Measurements

Creep-recovery testing was performed as previously described in a previous study [24], and the maximum creep compliance (*J_max_*), zero shear viscosity (*η*_0_), relative elastic part of the maximum creep compliance (*J_e_/J_max_*), and relative viscous part of the maximum creep compliance (*J_v_/J_max_*) were calculated using RHEOPLUS/32 version 3.21 software.

### 2.4. Texture Profile Analysis

TPA analysis was carried out based on our previous work [7]. Briefly, testing was conducted using a texture analyzer (Stable Micro Systems, London, UK) with a cylindrical probe (50 mm diameter). The instrument settings were as follows: auto 10 g trigger force, 3 mm/s pre-test speed, 3 mm/s post-test speed, 1 mm/s test speed, 70% strain, and 10 s time. Six biological replicates were tested from each group.

### 2.5. Microstructure Observation (SEM)

Before the microstructure observation, the thawed dough was freeze-dried for 72 h in a vacuum freezer. The freeze-dried samples were carefully broken up. A thin layer was then mounted on the copper sample holder with double-sided carbon tape and plated with gold particles [7]. The observation was conducted using S-570 Scanning Electron Microscopy (Hitachi, Tokyo, Japan). The instrument settings were as follows: 10 kV of acceleration voltage and 200× magnification of the image.

### 2.6. Statistical Analysis

Data analysis was performed using SPSS 22.0 software (IBM Corporation, Armonk, NY, USA). One-way ANOVA followed by Duncan’s multiple-range test was applied to analyze the differences between dough under different treatments. *p* < 0.05 was considered statistically significant. The general linear model (GLM) was performed and the contribution rate following the mean square value was calculated. All of graphs were drawn by OriginPro 2021b (Origin Lab Corporation, Northampton, MA, USA).

## 3. Results

### 3.1. Dynamic Rheological Properties of the Dough

#### 3.1.1. Dynamic Strain Sweep

The linear viscoelasticity region (*LVR*) limit is a parameter that can offer information related to the objectives’ relative strength of the junction zones and their relative resistance to flow [23]. The *LVR* values of the dough ranged from 0.23–0.33% (Table 2). Furthermore, the unfrozen fresh dough showed the highest *LVR*s. Long-term frozen storage produced dough with lower *LVR*s values and the dough frozen and stored for 30 days with microwave thawing (MT), which exhibited the lowest *LVR*s limits (*p < 0.05*), indicating a weak and fragile network for this dough. Additionally, there was no significant difference among the three thawing groups with 1 day of frozen storage, whereas a significant difference was obtained between MT and other thawing groups after 30 days of frozen storage. Thus, frozen storage time had a more obvious impact on the *LVR*s of frozen dough.

#### 3.1.2. Dynamic Frequency Sweep

The viscoelastic properties can be also reflected by G values as a function of angular frequency (ω). The storage modulus (*G*′) and loss modulus (*G*″) are the key indicators for evaluating the dough’s rheological characteristics, presenting the solid and liquid state of the dough. In this study, the *G*′ of the dough was higher than *G*″, indicating a solid state of dough. Compared with the unfrozen dough, which had the highest *G* values, the dough that had been frozen showed significantly low *G*′ and *G*″ values, suggesting both the frozen storage and thawing treatments had a negative impact on the rheological properties of frozen dough. Furthermore, the *G*′ value for both the dough frozen and stored 1 day and for 30 days showed significant differences between microwave thawing (MT) and other thawing groups (RT and CT). The frozen samples with refrigerator thawing and microwave thawing always showed the highest and lowest elasticity and viscosity in both the 1-day stored dough and 30-day stored dough, respectively, suggesting that microwave thawing created more obvious damage to the dough.

Additionally, based on the logarithmic plot of *G*′ = *K*′(*ω*)^*z*′^ [24], the relationship between *G*′ and frequency can be determined. The *K*′ value indicates the strength of the dough. Generally, a higher *K*′ value represents a strong strength of dough [23]. Meanwhile, the *z*′ value reflects the type of molecular interaction. *z*′ = 0 means a covalent linkage with a stable network structure, a higher *z*′ value indicates a fragile network structure in the dough. The imitative effect of the model can be reflected by the corresponding coefficients of determination (*R*^2^). The results showed that the *G*′ values of all of the dough were dependent on frequency *(R*^2^ > 0.99) (Table 2). Further, the *K*′ value ranged from 3.440 to 3.858, and the highest *K*′ value was found for the unfrozen dough, indicating the highest strength of the dough. Meanwhile, the *z*′ of all of the dough ranged from 0.245 to 0.293, suggesting the dough had a physical linkage with low stability in the network structure. The frozen storage and thawing treatment affected the *K*′ and *z*′ values differently. Among all of the frozen−thawed treatment dough, the dough stored for 30 days and then thawed using a microwave had the highest *K*′ value and the lowest *z*′ value, indicating a weak network structure in the dough. It is worth noting that the dough stored for 1 day had no significant difference in different thawing condition groups. However, after 30 days of frozen storage, both the *K*′ and *z*′ values using microwave thawing treatment showed notably different values compared with the other thawing groups. The results indicate that the dough in long-term frozen storage is more sensitive to thawing treatments.

#### 3.1.3. Creep and Recovery Measurements

In general, non-linear creep-recovery measurements were performed to evaluate the macrostructure properties of the dough. The maximum compliance (*J_max_*) could be used to evaluate the deformation capacity and rigidity of the dough during the creep phase. Higher compliance indicated lower dough hardness [23]. In this study, both the frozen storage time and thawing treatment had marked effects on *J_max_*, while their interaction effect was also significant. The *J_max_* of the frozen samples decreased significantly compared with the unfrozen dough, while the lowest compliance was observed in the samples stored for 30 days and then treated by microwave thawing (Table 3). For the dough stored only for 1 day, no notable difference between the three thawing groups was obtained. But, after 30 days of frozen storage, a significant difference among the different thawing groups was observed. This indicated that extended frozen storage increased the dough’s rigidity, and the dough was more vulnerable to thawing treatments. Additionally, zero shear viscosity (*η*_0_) indicated the flowability of dough during the creep phase. Frozen storage (S) and thawing treatment (T) had a significant impact on the *η*_0_ (*p* < 0.05). Compared to the frozen dough, the unfrozen fresh dough had a lower *η*_0_ value (Table 3). Regardless of frozen storage time, MT always produced a dough with the highest *η*_0_, followed by CT and RT, indicating it was harder for such MT-treated dough to maintain their shape, whereas it was easier for RT dough to retain their shape.

During the recovery phase, the relative elastic part of maximum creep compliance (*J_e_/J_max_*) and the relative viscosity part of maximum creep compliance (*J_v_/J_max_*) were obtained to evaluate the recovery capacity of the dough after deformation. Both the frozen storage time and thawing treatments affected these values significantly. The highest *J_e_/J_max_* value was shown by the unfrozen fresh dough, while the lowest was observed in the dough stored for 30 days and then treated using microwave thawing. This result indicated that frozen storage and thawing treatment reduced the recovery ability of the dough sample, and 30 days of frozen storage combined with the microwave thawing produced the dough with the worst recovery ability and inner structure. Moreover, the decreased *G*′, *J_max_*, and *J_e_/J_max_* values indicated that the dough with a long frozen storage time had a lower resistance to deformation, which may be because the mechanical action of ice crystals could lead to damage to the gluten matrix, resulting in more separated starch granules and a more ruptured gluten network [25]. Besides, the starch in the dough usually serves as a rigid filler, which helps to keep and form a stable network structure within the inner the dough [26]. During the frozen storage and thawing procedure, stress will be applied to the starch granules, which may cause a deterioration in granule integrity. As a result, it was difficult for dough to maintain their shape after frozen and thawing treatment.

### 3.2. Texture Profile Analysis (TPA) of Non-Fermented Dough

TPA parameters, including hardness, springiness, and adhesiveness, are the most common indicators for evaluating the dough’s rheological properties from the view of large deformation [27]. In this study, the frozen storage time (S) and thawing treatment (T) and their mutual effects (S × T) had a highly significant influence on dough hardness and springiness. Frozen storage combined with thawing treatments produced dough that were significantly harder than the unfrozen fresh dough (Table 4), and prolonged storage time further increased the hardness of the dough. Moreover, among the different thawing treatment groups, the dough samples treated with refrigerator thawing were softer, and the difference between RT and MT was significant, regardless of the frozen storage time.

Additionally, the values of the springiness and adhesiveness of the frozen dough decreased with extended storage time compared with the unfrozen fresh dough. There was no obvious difference between the dough different thawing treatments after 1-day frozen storage. However, microwave thawing produced dough with significantly lower springiness after 30 days of storage, indicating long-term storage exacerbated the difference between different thawing groups.

### 3.3. Microstructure

**Figure 1:**
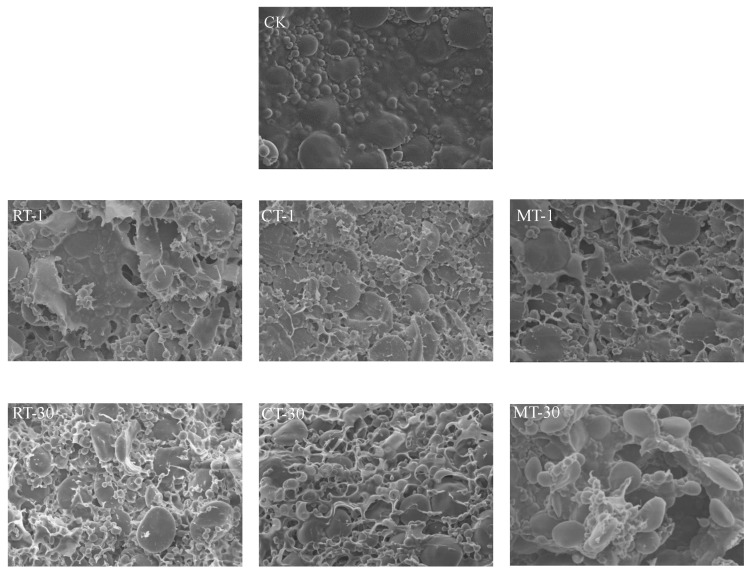
The scanning electron microscope images of dough (200×).

SEM images can provide information about dough’s structure from the view of the microstructure directly. Compared with the frozen dough, the fresh dough (CK) had a more flat, uniform inner surface with a denser gluten structure. Furthermore, the complete/contact starch granules were filled in the gluten network, which played a role in supporting the structure of the dough, and almost no obvious pores existed. In contrast, the inner structure of the dough changed notably. The angular voids gradually appeared in the gluten network, with part of the starch granules exposed (Figure 1). The 30-day frozen storage enlarged the pores and further deteriorated the gluten structure into pieces. Additionally, the protein structure of the 1-day frozen storage dough thawed with RT showed less structural destruction, with only some small pores, whereas the MT thawed dough was less homogeneous in structure with thinner strands and more pores. Moreover, for the dough stored for 30 days, thawing treatments had a significant impact on the inner structure and MT still resulted in maximum deterioration of the dough.

### 3.4. Multi-ANOVA Analysis

To evaluate the frozen storage time, thawing treatment, and their interaction effects on the rheological properties of the frozen−thawed dough, the general linear model (GLM) combined with an analysis of variance was performed (Table 5). The mean square value was used to compute the contribution rate for all factors. The higher the mean square value, the higher the contribution rate.

Frozen storage time and thawing treatments significantly influenced almost all of rheological parameters in the current study. However, their mutual effects were only significant for *z*′ value, hardness, and springiness. For all of the factors, the contribution order was S > T > S × T. Therefore, frozen storage time was the main factor that contributed largely, and thawing treatments also significantly contributed to most of the factors.

### 3.5. Relationship between TPA Properties and Rheological Properties

Pearson’s correlation analysis was performed to obtain the relationship between rheometer parameters and the texture profile analysis (Table 6). The results showed that hardness was positively correlated with z′ value, *η*_0_*,* and *J_v_/J_max_* (r = 0.89, 0.79, 0.735, respectively), while it was negatively correlated with *G*′, *K*′ value, *J_max_*, and *J_e_/J_max_*. In contrast, springiness was highly positively related to *G*′, *K*′ value, *J_max_*, and *J_e_/J_max_*. All of the parameters that are considered as indicators (springiness, *G*′, *K*′ value, *G*′, *K*′ value, *J_max_* and *J_e_/J_max_*) of an elastic dough with a better inner structure were positively correlated among themselves. All of these results indicate that both small viscoelastic parameters and large viscoelastic parameters played a key role in assessing the frozen dough′s rheological properties.

## 4. Discussion

In this study, frozen storage (S) was the dominant factor that contributed largely to all of the parameters. Previous studies have determined the variations in dough’s rheological properties during different frozen storage periods; however, it is still hard to produce a clear conclusion because of conflicting findings. For example, in this study, the hardness of frozen dough increased notably compared with the fresh one, supporting the findings of previous works [28,29]. However, the opposite results were also observed [30]. The reason for this might be related to the fact that when the type of dough was different and when the ingredients such as yeast, sugar, salt, and so on were added to dough formulations, the impact of frozen treatments on the properties of dough could be altered [3,4]. Additionally, *G*′ and springiness decreased significantly with increased storage time, and *G*′ was positively correlated with springiness, indicating a reduction in dough elasticity with the increased storage period. With increased storage time, *LVR*s and *J_max_* decreased, whereas *η*_0_ increased, suggesting a fragile structure for the 30-day frozen storage dough. The reason could be due to degradation of the gluten protein and disruption of the gluten cross-linking derived from ice crystallization and moisture redistribution. This hypothesis was also proven by the SEM results, in which the dough with a longer frozen storage time had a network structure with less continuity, more fractures, and more separation from starch granules.

The thawing treatment also had a significant impact on the rheological properties of frozen dough. Refrigerator thawing presented a more elastic and strong dough, regardless of the frozen storage period. In contrast, microwave thawing treatment presented a dough with a low stability and harder texture. The results also showed that microwave-thawed and refrigerator-thawed dough had the largest and smallest reduction in *G՛* and dough, respectively. On the one hand, refrigerator-thawed dough and environment chamber-thawed dough tended to show a uniform temperature distribution, but the microwave-thawed dough did not. Probably, during rheological measurement, this non-homogenous temperature distribution could lead to less uniform dough properties. On the other hand, the dough treated with microwave thawing was more likely to form fractures on the surface than the dough treated with other thawing conditions, which may also lead to increased hardness of the dough. Some studies reported that thawing treatments caused destabilization and structural damage to proteins in foods [31], which can also be responsible for the deterioration of dough’s rheological properties. Differently, Yang et al. [8] indicated microwave thawing led to a dough with the highest *G*′ value. However, the reports lacked the results of the dough structural parameters. The differences may be caused by the type of dough, the selection of the thawing parameters, and the measurement methods used on both sides. In the current study, we also found that frozen storage time was the dominant factor that contributed largely to all rheological parameters, followed by thawing treatments. For that matter, the long-term frozen storage aggravated the difference in the rheological properties among the three things treatments, indicating that after long-term storage, the dough was more sensitive to be influenced by thawing conditions. As mentioned above, during the frozen storage and thawing treatment, the ice crystallization, the recrystallization, and retrogradation of starch, as well as the depolymerization of gluten and water distribution, could have a negative impact on the structure and qualities of the dough. That is why the dough stored for 30 days was more vulnerable to thawing treatments, especially for microwave thawing.

Dough is a mixed system consisting of different ingredients such as protein, starch, and lipids. All of these substances play a role in stabilizing the dough’s structure. During cooking, a series of physicochemical reactions like polymerization reactions of the gluten protein, starch gelatinization, and lipid oxidation occur, which may influence the quality of the final products. Thus, further studies should be conducted to investigate the effects of the frozen storage and thawing conditions on the properties of the final products and to reveal if the viscoelastic properties of dough systems better correlate with the final cooked products’ quality. On this basis, small deformation testing combined with large deformation testing should be considered as a tool to predict the final frozen dough product’s quality.

## 5. Conclusions

Compared with unfrozen fresh dough, both the frozen storage time (S) and thawing treatment (T) influenced almost all indicators significantly. Meanwhile, their mutual effects (S × T) had a significant impact on the hardness and springiness values of the dough. Frozen storage time was the dominant factor resulting in the destruction of non-fermented dough compared with thawing treatments. Moreover, the short-term frozen storage dough and the long-term frozen stored dough had different responses to thawing treatment in terms of rheological properties. The difference between the three thawing treatment groups was exacerbated after 30 days of frozen storage. Moreover, the frozen dough thawed by the refrigerator and environment chamber had the overall best results. Long-term frozen storage combined with microwave thawing seriously destroyed the rheological properties, structural stability, and inner microstructure of the dough.

## Figures and Tables

**Table 1:** The quality of flour used in the experiment.

Moisture Content (%)	Protein Content (%)	Wet Gluten (%)	Ash Content (%)	Water Absorption (%)	Development Time (min)	Stability Time (min)	Maximum Resistance (BU)	Extensibility (mm)
13.1 ± 0.02	10.8 ± 0.04	32.6 ± 0.17	0.4 ± 0.01	65.8 ± 0.04	3.3 ± 0.12	2.2 ± 0.12	132 ± 2.60	211 ± 1.20

**Table 2:** Effect of thawing treatment on the *G*′ and *G*″ and the structural parameters of non-fermented dough frozen stored for 1 day and 30 days, respectively.

Frozen Storage Time (d)	Thawing Conditions	*LVR*s (%)	*G*′ (kPa)	*G″* (kPa)	*z*′	*K*′	*R* ^2^
CK		0.33 ± 0.03	6.88 ± 0.04	2.69 ± 0.08	0.249 ± 0.01	3.858 ± 0.061	0.995 ± 0.000
1	RT	0.33a ± 0.03	5.99a ± 0.09	2.40a ± 0.10	0.248a ± 0.00	3.790a ± 0.006	0.993 ± 0.007
	CT	0.30a ± 0.02	5.85ab ± 0.02	2.31a ± 0.20	0.245a ± 0.00	3.776a ± 0.028	0.994 ± 0.003
	MT	0.28a ± 0.02	5.34b ± 0.05	2.28a ± 0.10	0.258a ± 0.00	3.716a ± 0.040	0.991 ± 0.002
30	RT	0.28A ± 0.01	5.54A ± 0.05	2.54B ± 0.01	0.265B ± 0.00	3.674A ± 0.006	0.991 ± 0.005
	CT	0.28A ± 0.01	5.16B ± 0.02	2.49B ± 0.10	0.270AB ± 0.01	3.672A ± 0.008	0.992 ± 0.006
	MT	0.23B ± 0.00	4.86B ± 0.02	2.35B ± 0.07	0.293A ± 0.01	3.440B ± 0.043	0.994 ± 0.004

Note: a, b: Different mean values in the same column with different superscript letters differ significantly (*p* < 0.05) for the dough frozen and stored for 1 day. A, B: Different mean values in the same column with different superscript letters differ significantly (*p* < 0.05) for the dough frozen and stored for 30 days. RT, CT, and MT represent refrigeration thawing (4 °C), environment chamber thawing (25 °C, 85%RH), and microwave thawing (1000 W), respectively. The same is true in the following tables.

**Table 3:** Effects of thawing methods on the creep-recovery parameters of frozen dough stored for 1 day and 30 days, respectively.

Frozen Storage Time (d)	Thawing Treatments	Creep-Phase		Recovery-Phase	
		*J_max_*	*η*_0_	*J_e_/J_max_*	*J_v_/J_max_*
		10^−4^ Pa^−1^	10^5^ Pa^−1^	(%)	(%)
CK		37.2 ± 3.8	1.8 ± 0.1	52.7 ± 0.0	47.2 ± 4.1
1	RT	31.4a ± 0.0	2.1a ± 0.10	51.1a ± 0.6	48.8a ± 2.1
	CT	29.5a ± 2.7	2.3a ± 0.11	48.6a ± 2.1	50.7a ± 3.6
	MT	26.9a ± 0.0	2.8a ± 0.34	48.3a ± 0.6	51.7a ± 1.9
30	RT	18.3A ± 1.3	2.5B ± 0.33	46.4A ± 0.7	53.6A ± 2.0
	CT	13.7B ± 0.6	2.8B ± 0.14	45.1B ± 0.3	55.1A ± 1.5
	MT	8.8C ± 0.9	3.1A ± 0.03	42.9C ± 0.1	55.4A ± 2.0

Note: a: Different mean values in the same column with different superscript letters differ significantly (*p* < 0.05) for the dough frozen and stored for 1 day. A–C: Different mean values in the same column with different superscript letters differ significantly (*p* < 0.05) for the dough frozen and stored for 30 days. RT, CT, and MT represent refrigeration thawing (4 °C), environment chamber thawing (25 °C, 85%RH), and microwave thawing (1000 W), respectively.

**Table 4:** Effect of thawing treatments on TPA parameters of frozen non-fermented dough stored for 1 day and 30 days, respectively.

Frozen Storage Time (d)	Thawing Methods	Hardness (N)	Springiness	Adhesiveness (N s)
CK		106.11 ± 3.33	0.85 ± 0.07	465.78 ± 10.66
1	RT	126.22a ± 1.51	0.84a ± 0.00	512.73a ± 92.08
	CT	131.96a ± 5.52	0.84a ± 0.01	491.23a ± 10.71
	MT	225.71b ± 4.07	0.83a ± 0.06	471.97a ± 141.51
30	RT	267.0B ± 26.11	0.51A ± 0.0	359.0A ± 21.7
	CT	274.7B ± 20.69	0.48A ± 0.05	339.8A ± 8.8
	MT	307.4A ± 12.93	0.38B± 0.09	355.54A ± 1.6

Note: a, b: Different mean values in the same column with different superscript letters differ significantly (*p* < 0.05) for the dough frozen and stored for 1 day. A, B: Different mean values in the same column with different superscript letters differ significantly (*p* < 0.05) for the dough frozen and stored for 30 days. RT, CT, and MT represent refrigeration thawing (4 °C), environment chamber thawing (25 °C, 85%RH), and microwave thawing (1000 W), respectively.

**Table 5:** Multi-ANOVA variance analysis of the dough’s textural and rheological properties.

Source of Variation	Frozen Storage Time (S)	Thawing Treatment (T)	(S × T)	Error
df	1	2	2	6
*LVR*s	0.004 *	0.003 *	0.000	0
*G*′	8.58 × 10^5^ *	4.52 × 10^5^ **	0.18 × 10^5^	33,852
*G*”	5.32 × 10^4^	2.34 × 10^4^ *	0.34 × 10^4^	25,000.083
*z*′	0.002 **	0.001 *	7.65 × 10^−5^	2.76 × 10^−5^
*K*′	0.082 **	0.03 **	0.009 *	0.001
*J_max_*	7.36 × 10^−6^ **	4.95 × 10^−7^ **	6.27 × 10^−8^	3.63 × 10^−8^
*η*_0_	5.39 × 10^9^ *	4.39 × 10^9^ *	0.16 × 10^9^	0.71 × 10^9^
*J_e_*/*J_max_*	63.895 **	10.521 *	1.129	1.594
*J_v_*/*J_max_*	46.768 *	6.464	0.032	7.379
Hardness	4,660,115.114 **	572,728.769 **	91,845.983 *	5836.077
Springiness	0.48 **	0.007 **	0.004 *	0.003
Adhesiveness	5,314,297.02 *	467,335.108	242,172.446	556,093.543

Note: (df) Degree of freedom; (MS) Mean square, ** indicated highly statistically significant difference *(p* < 0.01), * indicated statistically significant difference *(p* < 0.05).

**Table 6:** Relationship within fundamental and empirical dough rheological properties.

	*G*′	*G”*	*z*′	*K*′	*J_max_*	*η*_0_	*J_e_/J_max_*	*J_v_/J_max_*	Hardness	Springiness	Adhesiveness
*LVR*s	−0.051	−0.405	−0.109	0.071	0.28	0.1	0.277	−0.379	−0.222	0.508 *	−0.356
*G*′		0.186	−0.888 **	0.861 **	0.855 **	−0.865 **	0.891 **	−0.781 **	−0.860 **	0.711 **	−0.665 *
*G”*			0.077	0.092	−0.221	0.012	0.035	−0.166	0.279	−0.419	0.409
*z*′				−0.946 **	−0.912 **	0.803 **	−0.843 **	0.631 *	0.890 **	−0.835 **	0.652 *
*K*′					0.861 **	−0.711 **	0.864 **	−0.616 *	−0.818 **	0.765 **	−0.525
*J_max_*						−0.766 **	0.947 **	−0.760**	−0.925 **	0.962 **	−0.749 **
*η*_0_							−0.729 **	0.565	0.790 **	−0.634 *	0.713 **
*J_e_/J_max_*								−0.866 **	−0.890 **	0.877 **	−0.762 **
*J_v_/J_max_*									0.735 **	−0.727 **	0.755 **
Hardness										−0.879 **	0.846 **
Springiness											−0.763 **

Note: ** indicated highly statistically significant difference (*p* < 0.01), * indicated statistically significant difference (*p* < 0.05).

## Data Availability

Data are contained within the article.
